# Hypertension, sleep quality, depression, and cognitive function in elderly: A cross-sectional study

**DOI:** 10.3389/fnagi.2023.1051298

**Published:** 2023-02-07

**Authors:** Jiajie Chen, Xi Chen, Ruxue Mao, Yu Fu, Qin Chen, Cuntai Zhang, Kai Zheng

**Affiliations:** Department of Geriatrics, Tongji Hospital, Tongji Medical College, Huazhong University of Science and Technology, Wuhan, China

**Keywords:** hypertension, sleep quality, depression, cognitive function, mediating effects

## Abstract

**Background:**

Hypertension, sleep disorders, and depression are highly prevalent in the elderly population and are all associated with cognitive impairment, but the role that sleep quality and depression play in the association between hypertension and cognitive impairment is unclear. The aim of this study was to investigate whether sleep quality and depression have a mediating role in the association between hypertension and cognitive impairment.

**Methods:**

A cross-sectional study was conducted to collect data from the Tongji Hospital Comprehensive Geriatric Assessment Database. Sleep quality, depression and cognitive function were measured by the Pittsburgh Sleep Quality Index (PSQI), the Geriatric Depression Scale (GDS-15) and the Mini-Mental State Examination (MMSE), respectively. Correlation analysis, regression analysis and Bootstrap analysis were used to examine correlations between key variables and mediating effects of sleep quality and depression. Adjustments for multiple comparisons were performed using Benjamini-Hochberg adjustment for multiple testing.

**Results:**

A total of 827 participants were included, hypertension was present in 68.3% of the sample. After correcting for covariates, hypertensive patients aged 65 years or older had worse cognitive function, poorer-sleep quality and higher levels of depression. Sleep quality was significantly negatively associated with depression and cognitive function, while depression was negatively associated with cognitive function. Mediation analysis revealed that hypertension can affect cognitive function in older adults through a single mediating effect of sleep quality and depression and a chain mediating effect of sleep quality and depression.

**Conclusion:**

This study found that sleep quality and depression can mediate the relationship between hypertension and cognitive function in elderly. Enhanced supervision of sleep quality and depression in elderly patients with hypertension may be beneficial in maintaining cognitive function.

## Introduction

Hypertension is a highly prevalent disease affecting two-thirds of adults over 65 years old (Mills et al., 2016). It is associated with a significantly increased risk of major adverse cardiovascular and cerebrovascular events and death, including heart disease, stroke, renal failure, and cognitive impairment (Oliveros et al., 2020; Heizhati et al., 2021). Numerous studies have found that hypertension is associated with increased incidence of cognitive decline, vascular cognitive impairment, and Alzheimer’s disease (Chudiak et al., 2018; Ungvari et al., 2021). But in a cohort study with an average age of 75 years, no association was found between hypertension and dementia (Shah et al., 2006). In addition, a protective effect of hypertension on cognitive function was found in a cohort study of participants over 90 years (Corrada et al., 2017).

In addition to increasing the incidence of cardiovascular events, hypertension is often associated with somatic symptoms, lower quality of life, and role impairment (He, 2016). The headache, chest pain, and dizziness of hypertension often cause poor sleep quality (Saccò et al., 2013; Rabner et al., 2018). A large population-based study conducted in China found that hypertensive patients had poorer sleep quality than healthy people (Liu et al., 2016). In addition to this, hypertension is also associated with psychological problems and depression (Cramer et al., 2020; Hamam et al., 2020), and the prevalence of depression in adults with hypertension was 37.8% (Asmare et al., 2022). Among them, older hypertensive patients (aged 50 years and above) have a higher chance of depression, roughly twice as much as younger hypertensive patients (aged 18–49 years; Boima et al., 2020). However, the sleep quality and emotional state of many patients with hypertension have not been paid enough attention by doctors and patients themselves.

One third of human life is spent in sleep, and too long or too short sleep duration increases the risk of cardiovascular events and death (Wang et al., 2019). The prevalence of sleep disorders increases with age, thereby affecting a variety of neurological functions including cognitive function (Bradley et al., 2020). Poor sleep quality is an important symptom of sleep disorders (Fabbri et al., 2021). Studies found that poor sleep quality would aggravate the decline of subjective cognitive ability and increase the risk of dementia (Li et al., 2021; Xu et al., 2021). There is also a relationship between sleep quality and depression, and the link may be bidirectional (Komada et al., 2011; Li et al., 2018). And the relationship between depression and cognitive impairment in the elderly has been reported in several studies (Camacho-Conde and Galán-López, 2020; Chow et al., 2022).

Therefore, we aimed to determine the relationship between hypertension and cognitive function in adults over 50 years of age, and specifically analyze different age groups, and assess whether sleep quality and depression are mediating factors. This may provide a reference for improving the cognitive dysfunction of patients with hypertension and poor sleep quality or depression.

## Materials and methods

### Study design and population

This cross-sectional study was conducted between October 2019 and August 2022. Data were collected from the Comprehensive Geriatric Assessment Database of Tongji Hospital, Tongji Medical College, Huazhong University of Science and Technology, in China, whose ethics committee approved the study (TJ-IBR20220731). Inclusion criteria were as follows: (1) age 50 or older, (2) complete data on sleep quality, depression and cognitive function. Exclusion criteria were as follows: (1) brain tumors or mental illnesses including schizophrenia, organic psychosis and anxiety, (2) medical records were incomplete.

### Hypertension assessment

Hypertension was previously diagnosed by a physician as high blood pressure or systolic blood pressure ≥140 mm Hg or diastolic blood pressure ≥90 mm Hg (Jones et al., 2020). To measure blood pressure, participants remained seated and rested for at least 5 min, and then blood pressure was measured three times, each 2 min apart. The last measurement was recorded as the average of the second and third measurements (Lima-Costa et al., 2018).

### Cognitive assessment

Cognitive function was assessed by Mini-Mental State Examination (MMSE). MMSE consists of 30 individual items in eight dimensions (time orientation, place orientation, concentration, attention and calculation, recall, naming, comprehension/executive function, visuospatial skills), with a total score ranging from 0 to 30 (Jia et al., 2021). A higher score indicates better cognitive function. The diagnosis of cognitive impairment based on MMSE are different for people with different education levels. MMSE ≤19 for illiterate individuals, ≤22 participants with primary education, and ≤26 for those with middle school education and above was considered to be mild cognition impairment (MCI; Jia et al., 2021). The diagnostic criteria for dementia are according to the Diagnostic and Statistical Manual of Mental Disorders, Fourth Edition (DSM-IV; Creavin et al., 2016).

### Sleep quality

The Pittsburgh Sleep Quality Index (PSQI) was used to assess the quality of sleep during the past month. PSQI consists of 19 individual items in seven dimensions (subjective sleep quality, sleep latency, sleep duration, habitual sleep efficiency, sleep disturbances, use of sleep medication, and daytime dysfunction), each with a score of 0–3. The total score of PSQI is between 0 and 21, higher scores indicate poorer sleep quality (Xu et al., 2021). Mostly, scores >5 are considered as poor self-reported sleep quality (Buysse et al., 1989).

### Depression

The Geriatric Depression Scale (GDS-15) was used to assess depression. The GDS-15 asks participants to answer 15 yes/no questions related to depressive symptoms. The total score ranges from 0 to 15, with a score of 5 or more being considered a symptom of depression, with higher scores representing more severe depressive symptoms (Chen et al., 2021).

### Covariates

In the present study, we included as covariates confounding factors that may influence the relationship between hypertension and cognitive function. Among these, information on demographic and social characteristics included age, gender, and education level (primary school or below, middle school, high school, and college or above). Information on participants’ cases was also reviewed to collect information on whether participants had comorbid diabetes, stroke, coronary heart disease (CHD), and hyperlipidemia. Body Mass Index (BMI) = weight (in kg)/height^2^ (in m^2^). Smoking was defined as current smoking of at least one cigarette per day for 6 months or more. Drinking was defined as alcohol consumption at least once per week.

### Statistical analysis

We used unpaired *t*-test for continuous variables and *χ*^2^ test for categorical variables to compare participants with and without hypertension. Given that age-specific effects of hypertension on cognitive function may differ, we stratified our analyses by two age groups (aged 50–64 years, aged 65 years or older). Pearson correlation analysis and Spearman correlation analysis were used to explore the associations between hypertension, sleep quality, depression, and cognitive function. Linear regression models were used to investigate the association between hypertension, sleep quality, depression and cognitive function. We showed the beta coefficients for three nested models: (1) unadjusted; (2) adjusted for sociodemographic factors (age, sex, and education); (3) fully adjusted, including sociodemographic factors, diabetes, stroke, CHD, hyperlipidemia, BMI, smoking, and drinking. Before analysis, PSQI, GDS-15 and MMSE were standardized (Z score). The false discovery rates were adjusted by Benjamini-Hochberg, where *p* < 0.05 was the cutoff value for significance of coefficients of independent variables. All statistical analyses were performed using IBM SPSS V24.0 software (SPSS Inc., Chicago, IL, United States), and all tests were two-sided with significance level set at 0.05 (two-tailed).

Based on the results of the correlation analysis, we specifically examined the mediating effect of sleep quality and depression on the association between hypertension and cognitive function. The bias-corrected Bootstrap method in the PROCESS 3.5 Procedure for SPSS was used to explore a multiple mediation model, which was developed by Hayes (2013).

## Results

**Figure 1:**
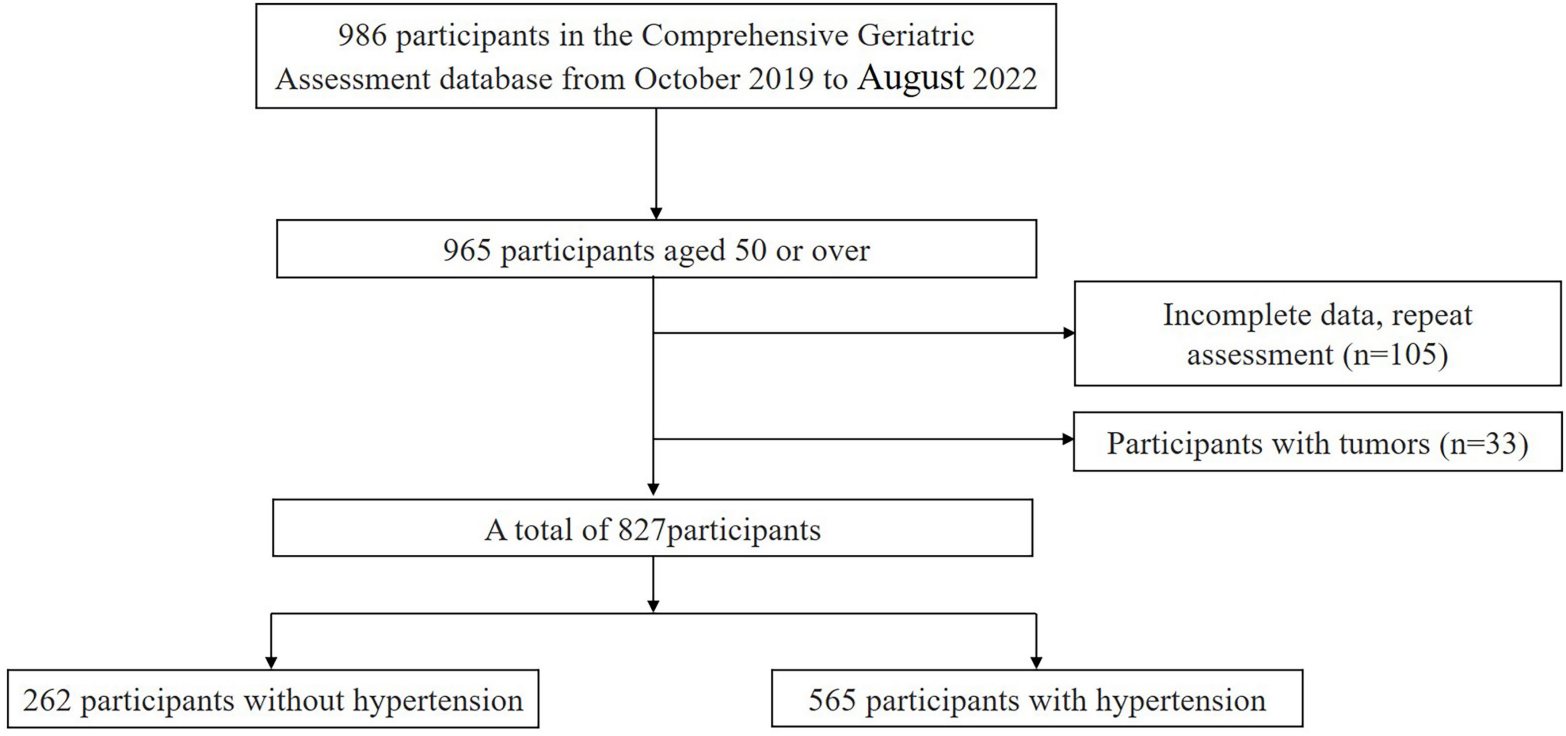
Participant inclusion flow chart.

A total of 982 participants were searched in the database, and 827 participants were finally included for analysis according to the inclusion and exclusion criteria, as described in Figure 1. The sociodemographic, anthropometric, lifestyle factors, sleep quality, depression, and cognitive function of participants grouped by hypertension status were shown in Table 1. In the whole sample, the average age of the subjects was 77.18 ± 10.78 years old, and 45.0% of them were women. Hypertension was present in 68.3% of the sample. In both age groups, the prevalence of hypertension was higher in people aged 65 years or older (73.5% vs. 36.0%). The MMSE score of the total participants was 23.30 ± 7.09, PSQI score was 7.74 ± 4.50, and GDS-15 score was 5.01 ± 3.84. In these two age groups, patients with hypertension have poorer cognitive function, more smokers, higher BMI, and higher stroke prevalence. Compared with participants aged 50–64, among people aged 65 or older, patients with hypertension drank and smoked more, had higher BMI, lower education level, higher prevalence of diabetes, stroke, coronary heart disease, hyperlipidemia, poorer cognitive function, higher GDS-15 scores, and poorer sleep quality.

**Table 1:** Participant characteristics according to hypertension and stratified by age groups.

	Whole sample			Adults aged 50-64 years			Adults aged 65 years or older		
	No (*n* = 262)	Yes (*n* = 565)	*p*	No (*n* = 73)	Yes (*n* = 41)	*p*	No (*n* = 189)	Yes (*n* = 524)	*p*
MMSE, mean (SD)	26.50 ± 4.35	21.82 ± 7.62	<0.001	27.38 ± 3.46	26.12 ± 4.24	0.09	26.15 ± 4.61	21.49 ± 7.72	<0.001
Age (years), mean (SD)	72.23 ± 11.04	79.47 ± 9.85	<0.001	59.00 ± 3.85	59.07 ± 4.25	0.93	77.33 ± 8.33	81.07 ± 8.25	<0.001
Women, *n* (%)	128 (48.9)	244 (43.2)	0.13	41 (56.2)	18 (43.9)	0.21	87 (46.0)	226 (43.1)	0.49
Drinking, *n* (%)	19 (7.3)	88 (15.6)	0.001	8 (11.0)	4 (9.8)	0.84	11 (5.8)	84 (16.0)	<0.001
Smoking, *n* (%)	39 (14.9)	155 (27.4)	<0.001	11 (15.1)	13 (31.7)	0.04	28 (14.8)	142 (27.1)	0.001
BMI, mean (SD)	22.08 ± 3.31	23.17 ± 3.47	<0.001	22.12 ± 3.34	24.35 ± 3.98	0.002	22.06 ± 3.31	23.08 ± 3.42	<0.001
Education, *n* (%)			0.001			0.06			0.008
Primary school or below	50 (19.0)	106 (18.8)		2 (2.7)	4 (9.8)		48 (25.4)	102 (19.5)	
Middle school	44 (16.7)	165 (29.3)		12 (16.4)	12 (29.3)		32 (16.9)	153 (29.2)	
High school	65 (24.7)	100 (17.7)		23 (31.5)	6 (14.6)		42 (22.2)	94 (17.9)	
University or over	104 (39.5)	193 (34.2)		36 (49.3)	19 (46.3)		67 (35.4)	175 (33.4)	
Diabetes, *n* (%)	32 (12.2)	125 (22.1)	0.001	8 (11.0)	7 (17.1)	0.35	24 (12.7)	118 (22.5)	0.004
Stroke, *n* (%)	30 (11.5)	164 (29.0)	<0.001	4 (5.5)	13 (31.7)	<0.001	26 (13.8)	151 (28.8)	<0.001
Coronary heart disease, *n* (%)	12 (4.6)	121 (21.4)	<0.001	3 (4.1)	3 (7.3)	0.46	9 (4.8)	118 (22.5)	<0.001
Hyperlipidemia, *n* (%)	34 (13.0)	128 (22.7)	0.001	7 (9.6)	8 (19.5)	0.13	27 (14.3)	120 (22.9)	0.01
PSQI, mean (SD)	6.78 ± 3.92	8.19 ± 4.68	<0.001	6.23 ± 3.16	7.54 ± 4.69	0.12	6.99 ± 4.16	8.24 ± 4.68	0.001
GDS-15, mean (SD)	4.22 ± 3.76	5.38 ± 3.83	<0.001	4.19 ± 3.17	5.17 ± 3.71	0.14	4.23 ± 3.97	5.40 ± 3.84	<0.001

*p* compare participants with and without hypertension using the independent-samples *t*-test for continuous variables and the *χ*^2^ test for categorical variables.

The correlation between key variables is shown in Table 2. Hypertension was positively associated with poor sleep quality and depression, but negatively associated with cognitive function. Depression was positively associated with poor sleep quality and significantly negatively associated with cognitive function. There was a significant negative association between poor sleep quality and cognitive function. The correlation was evident mainly among participants over 65 years or older. Table 3 shows the regression analysis of sleep quality and depression between hypertension and cognitive function. The total and direct effects of hypertension on cognitive function were significant in participants over 65 years of age. However, in people aged 50–64, there was no significant correlation between the above variables. The above conclusions were obtained after adjusting the covariates. The results of linear regression between the covariates and MMSE are in Supplementary Table 1.

**Table 2:** The correlation among key variables.

	Whole sample				Adults aged 50–64 years				Adults aged 65 years or older			
	Hypertension	PSQI	GDS-15	MMSE	Hypertension	PSQI	GDS-15	MMSE	Hypertension	PSQI	GDS-15	MMSE
Hypertension	1				1				1			
PSQI	0.14^**^	1			0.1	1			0.12^**^	1		
GDS-15	0.17^**^	0.34^**^	1		0.12	0.24^*^	1		0.17^**^	0.35^**^	1	
MMSE	−0.36^**^	−0.20^**^	−0.22^**^	1	−0.18	0.04	−0.00	1	−0.33^**^	−0.20^**^	−0.23^**^	1

* *p* < 0.05.

** *p* < 0.01.

**Table 3:** Associations from multiple linear regression models of hypertension with sleep quality, depression and cognitive function.

	Result variable	Predictor variable	Unadjusted		Model 1		Model 2	
			*β* (95% CI)	*p*	*β* (95% CI)	*p*	*β* (95% CI)	*p*
Whole sample	MMSE	Hypertension	−0.31 (−0.37, −0.24)	<0.001	−0.21 (−0.27, −0.14)	<0.001	−0.14 (−0.19, −0.08)	<0.001
	PSQI	Hypertension	0.15 (0.08, 0.21)	<0.001	0.13 (0.06, 0.20)	<0.001	0.13 (0.06, 0.21)	0.001
	GDS-15	Hypertension	0.09 (0.03, 0.16)	0.005	0.09 (0.03, 0.16)	0.008	0.11 (0.04, 0.18)	0.004
		PSQI	0.32 (0.26, 0.39)	<0.001	0.32 (0.25, 0.38)	<0.001	0.31 (0.25, 0.38)	<0.001
	MMSE	Hypertension	−0.27 (−0.34, −0.21)	<0.001	−0.18 (−0.24, −0.12)	<0.001	−0.11 (−0.17, −0.06)	0.001
		PSQI	−0.11 (−0.18, −0.04)	0.002	−0.08 (−0.14, −0.02)	0.01	−0.08 (−0.13, −0.03)	0.004
		GDS-15	−0.15 (−0.21, −0.08)	<0.001	−0.13 (−0.19, −0.07)	<0.001	−0.09 (−0.15, −0.04)	0.001
Adults aged 50-64 years	MMSE	Hypertension	−0.16 (−0.18, 0.01)	0.15	−0.10 (−0.15, 0.04)	0.32	0.00 (−0.09, 0.10)	0.98
	PSQI	Hypertension	0.17 (−0.02, 0.29)	0.15	0.13 (−0.04, 0.26)	0.32	0.17 (−0.04, 0.31)	0.46
	GDS-15	Hypertension	0.10 (−0.07, 0.25)	0.38	0.11 (−0.07, 0.25)	0.32	0.14 (−0.06, 0.29)	0.46
		PSQI	0.22 (0.04, 0.42)	0.15	0.20 (0.02, 0.40)	0.2	0.16 (−0.03, 0.36)	0.46
	MMSE	Hypertension	−0.17 (−0.19, 0.01)	0.15	−0.12 (−0.16, 0.04)	0.32	−0.01 (−0.10, 0.09)	0.98
		PSQI	0.06 (−0.08, 0.16)	0.6	0.11 (−0.05, 0.19)	0.32	0.07 (−0.06, 0.15)	0.71
		GDS-15	0.01 (−0.11, 0.12)	0.95	−0.02 (−0.13, 0.11)	0.88	−0.00 (−0.11, 0.11)	0.98
Adults Aged 65 years or older	MMSE	Hypertension	−0.28 (−0.38, −0.23)	<0.001	−0.23 (−0.32, −0.18)	<0.001	−0.16 (−0.24, −0.11)	<0.001
	PSQI	Hypertension	0.12 (0.05, 0.21)	0.001	0.12 (0.04, 0.20)	0.003	0.12 (0.04, 0.21)	<0.001
	GDS-15	Hypertension	0.09 (0.02, 0.17)	0.01	0.09 (0.02, 0.17)	0.02	0.10 (0.03, 0.19)	0.006
		PSQI	0.33 (0.27, 0.40)	<0.001	0.33 (0.26, 0.40)	<0.001	0.32 (0.25, 0.39)	<0.001
	MMSE	Hypertension	−0.25 (−0.34, −0.19)	<0.001	−0.20 (−0.29, −0.15)	<0.001	−0.13 (−0.21, −0.08)	<0.001
		PSQI	−0.11 (−0.19, −0.04)	0.002	−0.10 (−0.17, −0.03)	0.005	−0.10 (−0.16, −0.04)	0.002
		GDS-15	−0.16 (−0.24, −0.09)	<0.001	−0.13 (−0.20, −0.06)	<0.001	−0.09 (−0.15, −0.03)	0.004

Estimates were calculated using linear regression analysis. Model 1 = adjusted for sociodemographic factors (age, sex and education). Model 2 = adjusted for sociodemographic factors + diabetes, stroke, coronary heart disease, hyperlipidemia, BMI, smoking and drinking. CI, credibility interval. A Benjamini-Hochberg adjustment was applied to the false discovery rate, where *p* < 0.05 was considered a statistically significant coefficient of the independent variable.

**Figure 2:**
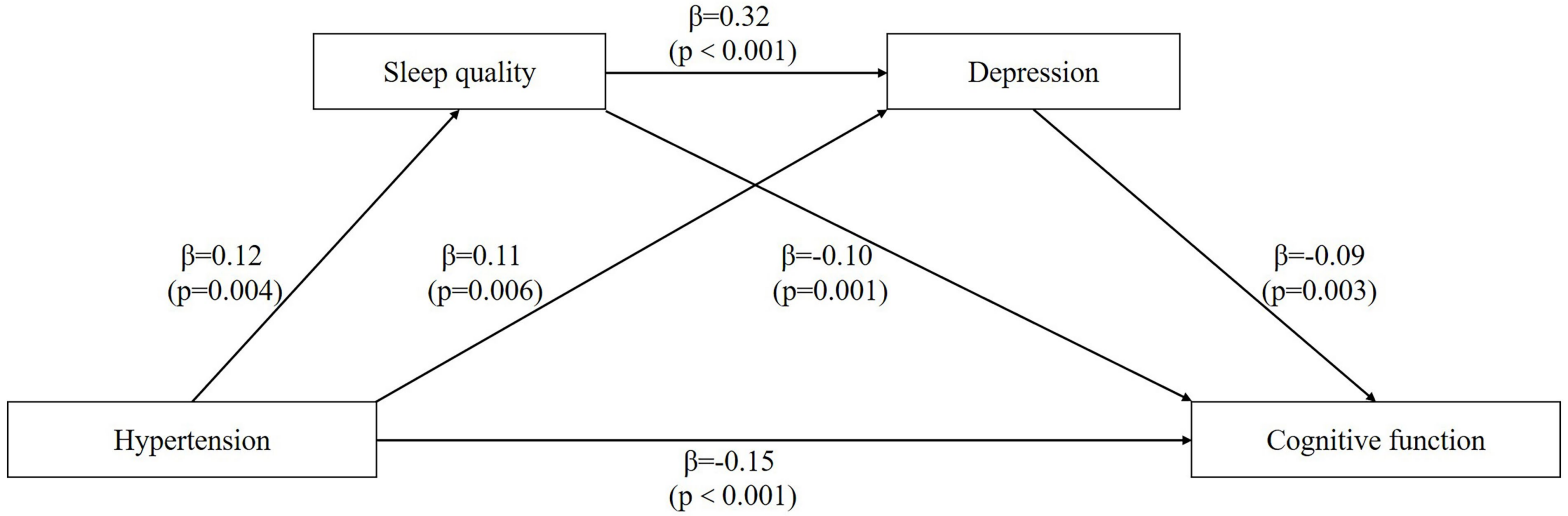
The chain mediation model. The chain mediation model shows the effects of hypertension, sleep quality, and depression on the cognitive function.

Table 4 shows the mediating effect values of sleep quality and depression between hypertension and cognitive function in participants aged 65 years or older. Figure 2 shows the chain mediation model between hypertension and cognitive function in participants aged 65 years or older. Sleep quality and depression had a significant mediating effect between hypertension and cognitive function, and the total mediating effect was −0.026. Specifically, the mediating effect consisted of three indirect effects: path 1 hypertension → sleep quality → cognitive function (−0.012), path 2 hypertension → depression → cognitive function (−0.01), and path 3 hypertension → sleep quality → depression → cognitive function (−0.004). The proportion of the three indirect effects of path 1, path 2 and path 3 was 7.02, 5.85 and 2.34%, respectively, and the 95% confidence interval did not contain zero, indicating that the three indirect effects were all significant. There was no significant difference among the three indirect effects. These results suggest that hypertension affects cognitive function not only through a single mediating effect of sleep quality and depression, but also through a chain mediating effect of sleep quality and depression in the elderly aged 65 years or older.

**Table 4:** Sleep quality and depression in the mediation effect analysis.

	Indirect effects	Boot SE	Boot LLCI	Boot ULCI	Relative mediation effect
Total indirect effect	−0.026	0.009	−0.044	−0.011	15.20%
Indirect effect 1	−0.012	0.006	−0.025	−0.003	7.02%
Indirect effect 2	−0.01	0.006	−0.023	−0.001	5.85%
Indirect effect 3	−0.004	0.002	−0.008	−0.001	2.34%
Compare 1	−0.002	0.008	−0.018	0.015	
Compare 2	−0.009	0.006	−0.021	0.001	
Compare 3	−0.006	0.005	−0.018	0.002	

The direct and indirect effects of hypertension on cognitive function are shown in this table. Boot SE, Boot LLCI, and Boot ULCI refer, respectively, to the standard error and the lower and upper limits of the 95% confidence interval of the indirect effects estimated by the percentile bootstrap method with deviation correction. Indirect effect1: hypertension→ sleep quality→ cognitive function; indirect effect 2: hypertension→ depression→ cognitive function; indirect effect 3: hypertension→ sleep quality→ depression→ cognitive function.

## Discussion

Hypertension, sleep disorders, depression, and cognitive impairment all have a high prevalence in older adults, and the prevalence all increases with age, and co-morbidity of these disorders is common (Ou et al., 2020; Vanek et al., 2020). Our study explored the association between hypertension and cognitive function in elderly and is the first time to analyze the role of sleep quality and depression in it. The findings suggest that hypertension was significantly negatively associated with cognitive function in participants aged 65 years or older and this association was partially mediated by sleep quality and depression.

In our study, the prevalence of hypertension in the elderly over 65 years or old was much higher than that in the middle-aged (50–64 years old), which has also been reported in previous studies (Tsimihodimos et al., 2018). We found that elderly with hypertension had worse cognitive function than those with normal blood pressure, which is consistent with previous findings. Vasilopoulos et al. (2012) reported no significant difference in cognitive function in hypertensive patients aged 51–60 years, while in the relationship between hypertension and cognitive function in the elderly, hypertension was found to have a negative effect on cognitive function in people aged 60–74 years (Qiu et al., 2005), and hypertension over 75 years patients showed a significant decrease in cognitive function (Streit et al., 2019). The possible mechanism for this is that as we age, the form of hypertension may change from systolic/diastolic hypertension to systolic hypertension and aortic atherosclerosis, which severely affects the blood supply to the brain and thus causes cognitive impairment (Boutouyrie et al., 2021).

The phenomenon of cognitive impairment in hypertensive patients can be partially explained by sleep disturbances and depressive symptoms. First, numerous studies have found a much higher prevalence of poor sleep quality in hypertensive patients than in non-hypertensive patients (Lo et al., 2018; Li et al., 2020). The prevalence of poor sleep quality in hypertensive patients was 35.5%, of which high diastolic blood pressure and lack of exercise were independent predictors of poor sleep quality (Birhanu et al., 2021). Sleep is essential for good health, and sleep benefits the consolidation of memory (Westermann et al., 2015), while sleep deprivation may cause brain dysfunction and lead to cognitive impairment (Porter et al., 2015). The formation of new memories in the hippocampus depends on undisturbed sleep, either before or after the initial encoding of potential memories (Mander et al., 2017). In a large cohort study of older Chinese adults, lower habitual sleep efficiency was associated with a higher risk of memory impairment and poorer cognitive function (Ma X. Q. et al., 2019). Hypertension can not only directly impair cognitive function in the elderly, but also indirectly by reducing sleep quality.

Several studies have reported that hypertensive patients are more likely to experience depressive symptoms (Li et al., 2015; Ademola et al., 2019). Hypertensive patients often suffer from psychological distress due to antihypertensive medication side effects, decreased quality of life, and health impairment (Song et al., 2018). Recurrent depressive symptoms can lead to progressive hippocampal atrophy, which impairs memory function (Sheline et al., 2019). Due to the excessive activation of astrocytes and microglia, the level of inflammation in the hippocampus of depression model mice increased, and the cognitive function was impaired (Santos et al., 2016; Zhang et al., 2020). In population studies, depressive symptoms predict poorer memory scores and may be an early indicator of declining situational memory in older adults (Zahodne et al., 2014). A prospective study found that increased depressive symptoms at each life stage were associated with cognitive outcomes, and that depressive symptoms in later life were negatively associated with cognitive function and associated with a more rapid rate of cognitive decline (Mirza et al., 2016). Therefore, depression may play an important role in the impairment of cognitive function caused by hypertension.

The correlation between depressive symptoms and poor sleep quality in the elderly has been reported several times (Hu et al., 2020; Hsu et al., 2021). In animal experiments, chronic sleep deprivation induced depression-like behaviors in rat (Ma W. et al., 2019). Sleep deprivation in mice induces hippocampal neuroinflammation, which is a risk factor for depression (Kang et al., 2021). Sleep deprivation over-activated microglia in the mouse brain and causes a decrease in the level of anti-inflammatory factors associated with the hippocampus, which may be a possible cause of its depression (Ahmed et al., 2021). Among college students, as sleep quality deteriorates, the level of depression also increases., and the risk of depressive symptoms in students with poor sleep quality was 3.28 times (Çelik et al., 2019). Similarly, adolescent females exposed to stress such as sleep disruption are prone to hypothalamic–pituitary–adrenal sensitization which contributes to the development of mood disorders, such as depression (Murack et al., 2021). Of the 162 sleep related functional connections found in the human connectome study, 39 were also associated with depression scores (Cheng et al., 2018), which may partially explain the association between depression and poor sleep quality.

This study found the relationship between hypertension and cognitive impairment in people over 65 years or older can be partially mediated by sleep quality and depression, which suggests that attention should be paid to screening for sleep quality and depressive symptoms in elderly hypertensive patients, strengthening the diagnosis and treatment of sleep disorders and depression in the elderly, and improving the perception of mood disorders in elderly hypertensive patients, which may help to reduce the cognitive impairment and cardiovascular burden in patients.

Our study also has some limitations. This study was a cross-sectional study and could not establish a causal relationship between hypertension and cognitive function. Therefore, the conclusion of this study is relatively less reliable than that of a cohort study. On the basis of this study, more in-depth cohort study should be conducted to further verify our conclusion after follow-up of the included patients. In terms of basic experiments, the specific mechanism of this mediating effect may be revealed by detecting depression and cognitive function in hypertensive model mice after sleep deprivation. Second, no specific classification of hypertension was made in this study, and also the small sample size may cause bias in the results.

## Conclusion

In conclusion, the results of this study suggest that sleep quality and depression may partially mediate the relationship between hypertension and cognitive function in elderly over 65 years. Importantly, sleep quality and depression status can be reversed by intervention, which suggests that strengthening the supervision of sleep quality and depression in elderly hypertensive patients is of great significance for the prevention of cognitive dysfunction.

## Data availability statement

The raw data supporting the conclusions of this article will be made available by the authors, without undue reservation.

## Ethics statement

This study was approved by the ethics committee of the Huazhong University of Science and Technology, China (TJ-IBR20220731). Written informed consent was not required in accordance with local and institutional legislation.

## Author contributions

KZ conceived and supervised the study, wrote the commentary and edited it. CZ conceptualized and supervised the study. JC analyzed the data and wrote the original draft. XC collected the data and wrote the original draft. RM and YF curated data. QC edited the manuscript. All authors contributed to the article and approved the submitted version.

## Funding

This research was funded by the Fundamental Research Funds for the Central Universities (item number: 2019kfyXKJC055), and the National Key Research and Development Program of China (Project No. 2019YFC2004805 and 2020YFC2004800).

## Conflict of interest

The authors declare that the research was conducted in the absence of any commercial or financial relationships that could be construed as a potential conflict of interest.

## Publisher’s note

All claims expressed in this article are solely those of the authors and do not necessarily represent those of their affiliated organizations, or those of the publisher, the editors and the reviewers. Any product that may be evaluated in this article, or claim that may be made by its manufacturer, is not guaranteed or endorsed by the publisher.
